# Pulmonary lipiodol embolism following transcatheter arterial chemoembolization

**DOI:** 10.1002/rcr2.678

**Published:** 2020-10-22

**Authors:** Tomohiro Moriya, Naoki Kawakami, Yoko Wakai, Hiroaki Saito, Kazuhito Saito

**Affiliations:** ^1^ Department of Respiratory Medicine Tsuchiura Kyodo General Hospital Tsuchiura Japan

**Keywords:** Computed tomography, inferior phrenic artery, pulmonary lipiodol embolism, transcatheter arterial chemoembolization

## Abstract

Computed tomography of pulmonary lipiodol embolism reveals high‐density areas in the lung field or intrapulmonary blood vessels. One of the risk factors of lipiodol embolism is embolization of the inferior phrenic artery.

## Graphical abstract

One of the complications of transcatheter arterial chemoembolization (TACE) is pulmonary embolism. We present the computed tomography findings of pulmonary embolism in a 70‐year‐old patient who underwent TACE for recurrent hepatocellular carcinoma.

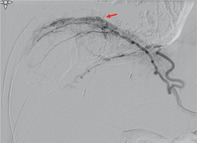

## Clinical Image

**Figure 1:**
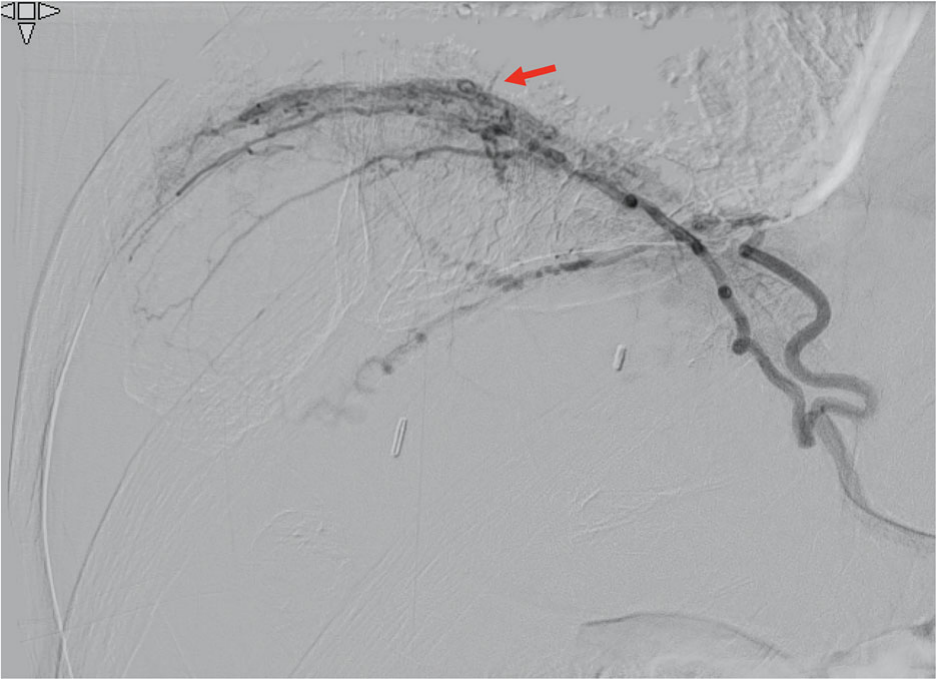
A perspective image reveals a branch running cephalad over the diaphragm from the right inferior phrenic artery.

**Figure 2:**
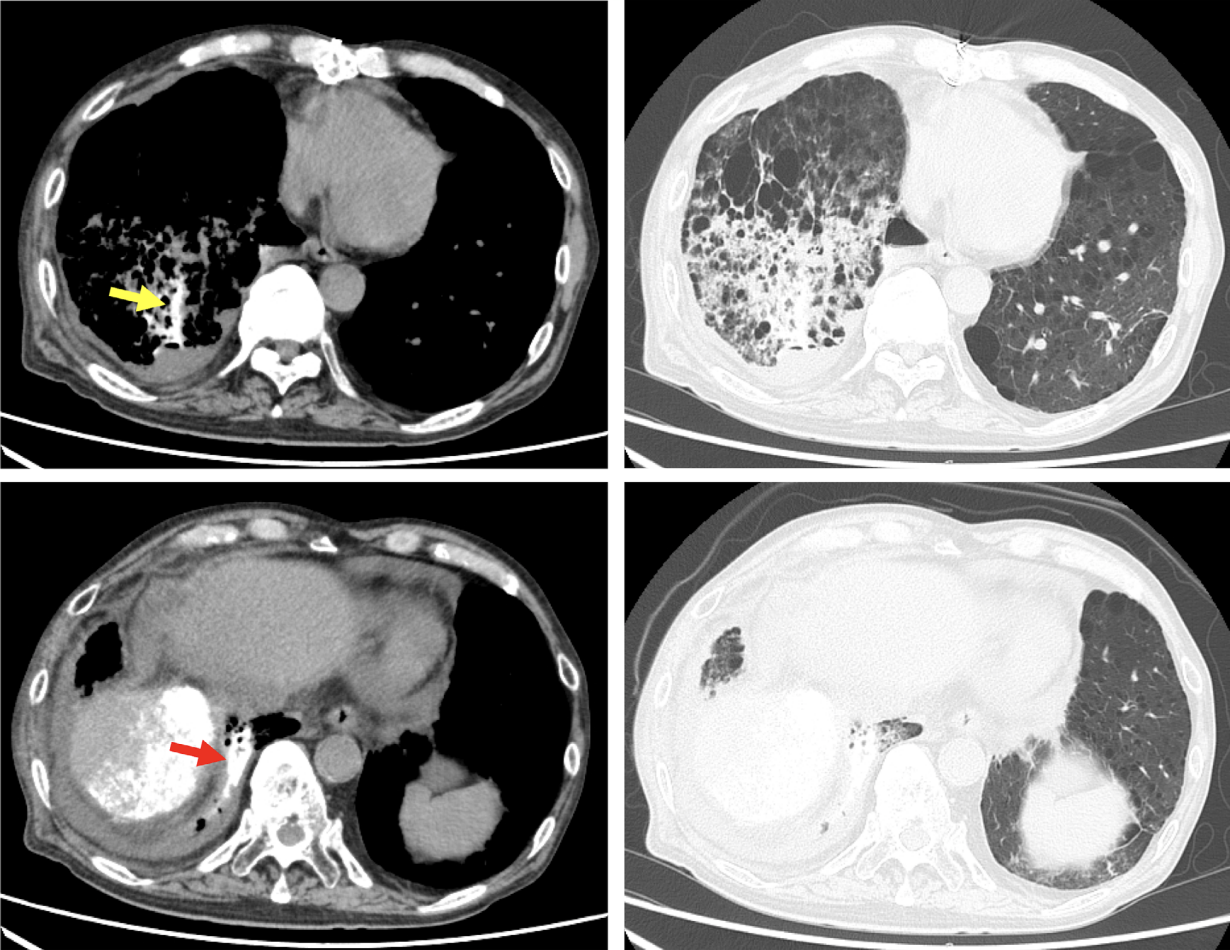
Consolidation was observed in the right lower lobe on non‐contrast computed tomography. A part of the consolidation (red arrow) and a pulmonary vein (V9, yellow arrow) had extremely high density.

A 70‐year‐old male with a history of pulmonary emphysema, pneumothorax, and chronic kidney disease developed hepatocellular carcinoma four years ago and underwent transcatheter arterial chemoembolization (TACE) thrice. He developed recurrence of the carcinoma in S7 segment (size: 2.5 cm) and underwent TACE for the fourth time. The right inferior phrenic artery was embolized using lipiodol, which migrated into the right lung and was confirmed in a perspective image. He developed fever the next day and, subsequently, respiratory failure. Non‐contrast computed tomography (CT) revealed consolidation in the right lower lobe and high density in the pulmonary vein (V9). A diagnosis of pulmonary lipiodol embolism was established based on operative findings during TACE and CT findings. Antibiotics and corticosteroids were administered. Pulmonary lipiodol embolism following TACE is a rare complication. Xu et al. reported that 2.3% of patients who underwent percutaneous TACE developed pulmonary oily embolism [1], the risk factors of which include the dose of the iodized oil, presence of an arteriovenous shunt, and trans‐inferior phrenic artery embolization [2]. In this patient, we had embolized the right inferior phrenic artery (Figs 1, 2).

### Disclosure Statement

Appropriate written informed consent was obtained for publication of this case report and accompanying images.
